# Heterogeneous Tumor-Immune Microenvironments among Differentially Growing Metastases in an Ovarian Cancer Patient

**DOI:** 10.1016/j.cell.2017.07.025

**Published:** 2017-08-24

**Authors:** Alejandro Jiménez-Sánchez, Danish Memon, Stephane Pourpe, Harini Veeraraghavan, Yanyun Li, Hebert Alberto Vargas, Michael B. Gill, Kay J. Park, Oliver Zivanovic, Jason Konner, Jacob Ricca, Dmitriy Zamarin, Tyler Walther, Carol Aghajanian, Jedd D. Wolchok, Evis Sala, Taha Merghoub, Alexandra Snyder, Martin L. Miller

**Affiliations:** 1 Cancer Research UK Cambridge Institute, University of Cambridge, Li Ka Shing Centre, Robinson Way, Cambridge CB2 0RE, UK; 2 European Molecular Biology Laboratory (EMBL), European Bioinformatics Institute, Wellcome Genome Campus, Hinxton, Cambridge CB10 1SD, UK; 3 Department of Medical Physics, Memorial Sloan Kettering Cancer Center, 1275 York Avenue, New York, NY 10065, USA; 4 Ludwig Collaborative/Swim Across America Laboratory, Memorial Sloan Kettering Cancer Center, 1275 York Avenue, New York, NY 10065, USA; 5 Department of Radiology, Memorial Sloan Kettering Cancer Center, 1275 York Avenue, New York, NY 10065, USA; 6 Department of Pathology, Memorial Sloan Kettering Cancer Center, 1275 York Avenue, New York, NY 10065, USA; 7 Gynecology Service, Department of Surgery, Memorial Sloan Kettering Cancer Center, 1275 York Avenue, New York, NY 10065, USA; 8 Gynecologic Medical Oncology Service, Department of Medicine, Memorial Sloan Kettering Cancer Center, 1275 York Avenue, New York, NY 10065, USA; 9 Parker Institute for Cancer Immunotherapy, Memorial Sloan Kettering Cancer Center, 1275 York Avenue, New York, NY 10065, USA; 10 Department of Medicine, Memorial Sloan Kettering Cancer Center, 1275 York Avenue, New York, NY 10065, USA; 11 Department of Medicine, Weill Cornell Medical College, New York, NY, USA; 12 Immunology and Microbial Pathogenesis Programs, Weill Cornell Graduate School of Medical Sciences, New York, NY 10065, USA

## Abstract

We present an exceptional case of a patient with high-grade serous ovarian cancer, treated with multiple chemotherapy regimens, who exhibited regression of some metastatic lesions with concomitant progression of other lesions during a treatment-free period. Using immunogenomic approaches, we found that progressing metastases were characterized by immune cell exclusion, whereas regressing and stable metastases were infiltrated by CD8^+^ and CD4^+^ T cells and exhibited oligoclonal expansion of specific T cell subsets. We also detected CD8^+^ T cell reactivity against predicted neoepitopes after isolation of cells from a blood sample taken almost 3 years after the tumors were resected. These findings suggest that multiple distinct tumor immune microenvironments co-exist within a single individual and may explain in part the heterogeneous fates of metastatic lesions often observed in the clinic post-therapy.

**Video Abstract:**

## Highlights

• Differential progression of metastases during off-treatment period.
• Coexistence of distinct tumor-immune microenvironments within the same individual.
• Tumor regression and progression correlated with T cell infiltration and exclusion.
• Clonal neoepitopes elicited reactivity of circulating CD8^+^ T cells.

## Graphical Abstract

**Figure:**
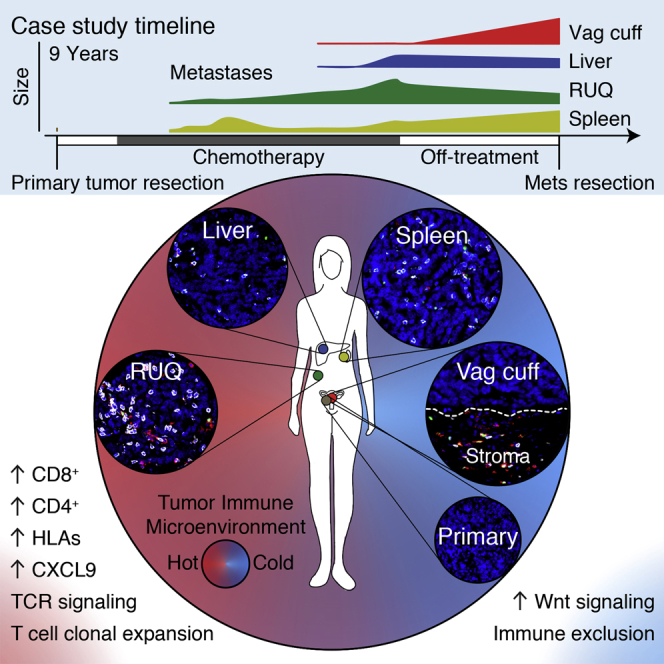

## Teaser

Distinct tumor immune microenvironments co-exist within a single individual and may help to explain the heterogeneous fates of metastatic lesions often observed post-therapy.

## Introduction

The majority of patients with ovarian cancer relapse despite appropriate surgery and chemotherapy (Bowtell et al., 2015, Cannistra, 2004). Ovarian cancer is characterized by a preponderance of DNA copy-number alterations and a modest somatic missense mutation burden (∼61 per exome) (Patch et al., 2015, Cancer Genome Atlas Research Network, 2011). Analysis of data from various cancer types studied by the Cancer Genome Atlas (TCGA) consortium, including ovarian cancer, has demonstrated that the number of somatic mutations and neoepitopes (peptides resulting from somatic non-silent mutations that are presented to the immune system) correlates with overall survival (Brown et al., 2014). Together with the observation that chemotherapy in some cases may trigger immune activation in ovarian cancer and other cancer types (Galluzzi et al., 2015, Gavalas et al., 2010, Pfirschke et al., 2016), this highlights the importance of investigating the host immune response in ovarian cancer. However, the interplay between somatic mutations, prior therapy, and the host immune response in this disease remains largely unknown.

Several studies of smaller cohorts of patients with metastatic ovarian cancer have found that primary and metastatic lesions exhibit heterogeneity at the genomic level (Bashashati et al., 2013, Lee et al., 2015, De Mattos-Arruda et al., 2014). Supporting these findings, functional magnetic resonance imaging (MRI)-based analysis has revealed that ovarian tumors and metastatic peritoneal implants are already phenotypically heterogeneous at diagnosis (Sala et al., 2012). As tumor heterogeneity increases the likelihood of presence of subclones able to escape the immune system (Bhang et al., 2015, Su et al., 2012, Turke et al., 2010), immune control may be particularly challenging in ovarian cancer due to extensive heterogeneity and the low number of potential mutation-derived epitopes.

The clinical challenge of tumor heterogeneity has been demonstrated recently in the context of immunotherapy: patients with less heterogeneous tumors, and hence with more clonal neoepitopes, were more likely to respond to checkpoint-blockade immunotherapy than patients with heterogeneous tumors (McGranahan et al., 2016). Whether chemotherapy and the immune system could work cooperatively is also being explored. In some settings, chemotherapy promotes immune cell homeostasis and activation (Carson et al., 2004, Gavalas et al., 2010, Pfirschke et al., 2016), tumor antigen release (Zitvogel et al., 2008), and decreased numbers of myeloid-derived suppressor cells in the tumor microenvironment (Suzuki et al., 2005). Furthermore, effector T cells have recently been implicated to play a role in abrogating fibroblast-mediated chemoresistance in a mouse model of ovarian cancer (Wang et al., 2016). Despite these findings, a unified model describing the effect of chemotherapy on the tumor heterogeneity and immune-tumor interactions has not yet been reached. A critical step toward understanding the effect of chemotherapy on advanced metastatic diseases and the immune response in humans is to analyze intra-patient matched primary and metastatic tumors (Brabletz et al., 2013). The ability to perform such analyses has been limited by the fact that multiple tumor sites from a single patient with advanced disease are rarely concurrently sampled, mainly due to the lack of clinical indication.

Here we present a case study of a high-grade serous ovarian cancer patient whose different metastases exhibited concomitant regression and progression after treatment with multiple types of chemotherapy. We used whole-exome sequencing, RNA expression data, immunohistochemistry, neoepitope prediction, in situ T cell receptor sequencing of tumor-infiltrating immune cells, and T cell-neoepitope challenge assays with intracellular cytokine staining (ICS) to investigate the genetic, molecular, and cellular components that potentially underlie this differential growth. In this heavily chemotherapy-treated patient, immune cell infiltration with clonal expansion of T cells, but not mutation or neoepitope number, correlated with tumor progression/regression status. Our immunogenomic analysis paints a portrait that immune infiltration and activation are different in each tumor at 2 years post-chemotherapy. Inter-site immune heterogeneity represents an important clinical challenge in the development of treatment modalities to overcome intra-patient tumor heterogeneity.

## Results

**Figure 1:**
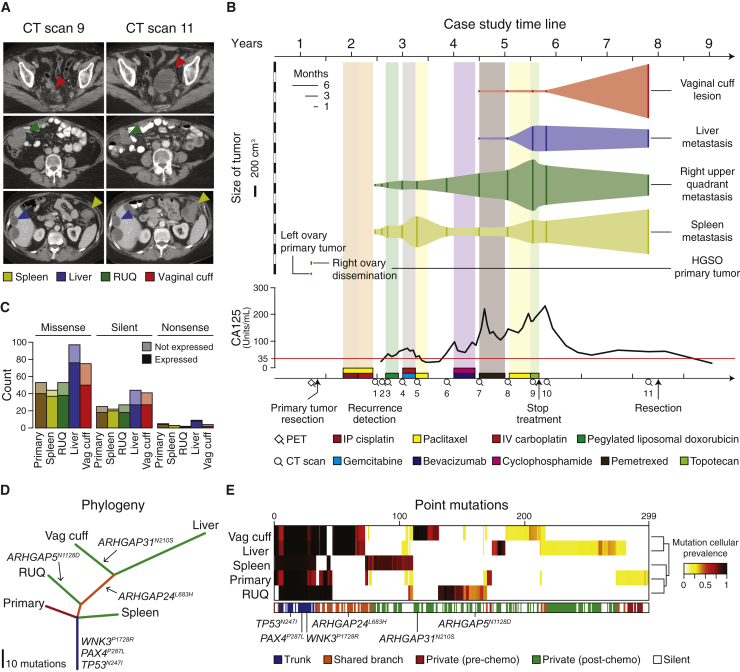
Metastatic Tumors Exhibit Heterogeneous Growth and Somatic Mutation Patterns after Multi-line Chemotherapy (A) Representative CT scans showing concomitant progression/regression of the different resected metastatic tumors. RUQ = right upper quadrant. “Spleen” refers to the tumor deposit adjacent to the spleen. (B) CT-based volume of metastatic lesions represented with the solid vertical lines and dynamics of quantified CA125 levels with the red line indicating the CA125 upper limit of normal (35 units/ml). The x axis at the bottom shows a timeline of therapeutic interventions and clinical follow up. (C) Number of missense, silent, and nonsense mutations. (D) The phylogenetic tree represents the relationship of the samples based on binary calls of non-silent point mutations (Table S1A). Length of the branches is proportional to the number of mutations. Potential driver mutations are indicated. (E) Hierarchical cluster analysis (Euclidean distance metric and “average” linkage method) of the cellular prevalence of point mutations (n = 299) estimated with PyClone (Roth et al., 2014) (Table S1B).

The patient presented here was diagnosed with stage IV high-grade serous ovarian adenocarcinoma, which typically exhibits a 5 year survival of 17% (National Cancer Institute, SEER Data Base), and underwent an optimal surgical debulking followed by paclitaxel combined with first cisplatin and then carboplatin. The patient experienced recurrence after 7 months, and during a period of 3 years she was treated with multiple regimens of chemotherapy with progression of disease after each therapy (Figures 1A and 1B). Her cancer was growing radiographically, and her CA125 was rising during treatment with topotecan when she then transitioned to best supportive care and was followed clinically with regular CA125 biomarker evaluation. After chemotherapy treatment was stopped, she experienced an atypical course: her CA125 decreased, and after 2 years of clinical follow up, CT scans showed evidence of differential growth of metastatic lesions including a new complex cystic mass in the vaginal cuff. Because of her long treatment-free interval and abdominal discomfort, she opted to undergo another debulking procedure, which found a substantial disease burden including tumor implants on the liver capsule, the splenic hilum, right upper quadrant (RUQ), and recto-vaginal space (Figures 1A and 1B). Samples of the primary and four metastatic tumors were submitted for whole-exome sequencing, microarray RNA quantification, staining for protein markers by immunofluorescence, and in situ T cell receptor sequencing.

### Phylogenetic Analysis of Somatic Mutations in Tumors

**Figure S1:**
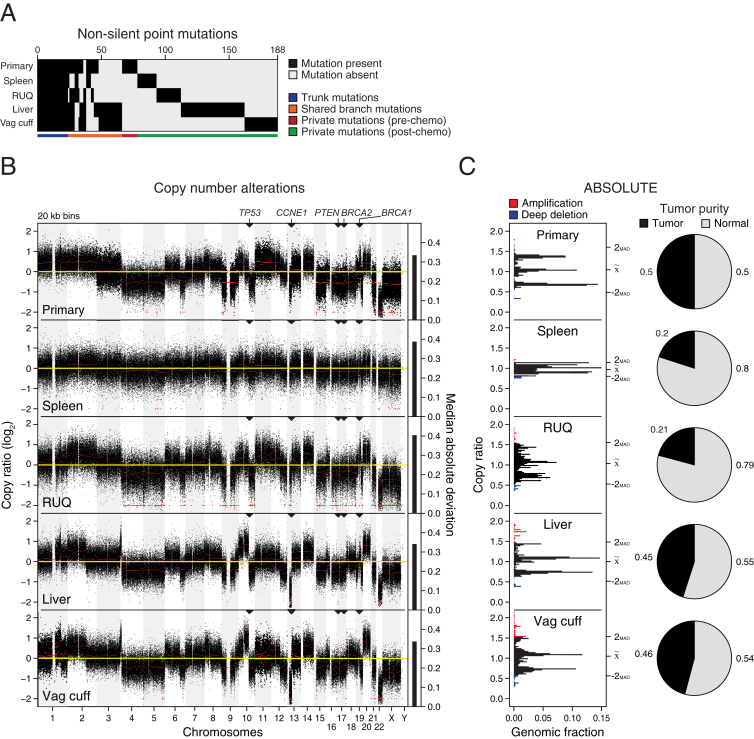
Non-Silent Somatic Mutations and Copy-Number Alterations, Related to Figures 1 and S2 and Table S1 (A) Binary matrix of present/absent non-silent point mutations (n = 188) used for the phylogeny tree reconstruction in Figure 1D (Table S1A). (B) Relative copy-number alterations inferred from WES data of the primary and metastatic samples using CopywriteR (Kuilman et al., 2015). (C) Relative copy number profiles and tumor purity inferred after ABSOLUTE (Carter et al., 2012) analysis. Amplified and deep deleted DNA segments were defined as copy number alterations with at least ± 2 median absolute deviations for each sample. MAD = median absolute deviation.

We performed whole-exome sequencing of normal blood and the resected samples to identify somatic mutations in the primary tumor and the metastases. Of all samples, we detected the highest mutation load in the liver and vaginal cuff metastases (Figure 1C). To infer the evolutionary relationship between the tumor samples, we used a binary presence/absence matrix of the non-silent mutations to perform a phylogenetic reconstruction based on the parsimony ratchet analysis method with branch lengths proportional to the number of non-silent mutations (Nixon, 1999, Schliep, 2011) (Figures 1D and S1A). The liver and vaginal cuff tumors were genetically more heterogeneous and harbored more mutations.

To estimate the proportion of cancer cells identified with a given mutation (cellular prevalence), we applied PyClone (Roth et al., 2014) using CopywriteR-inferred (Kuilman et al., 2015) DNA copy-number changes (Figures S1B and S1C) and ABSOLUTE-inferred (Carter et al., 2012) tumor purity and absolute copy numbers. As expected, truncal and shared mutations were generally clonal with high cellular prevalence, whereas private mutations had medium to low cellular prevalence indicating subclonal status (Figure 1E). Focusing on the specific genes that were mutated across all samples, we found among the truncal mutations potential oncogenic driver alterations, including *WNK3*^*P1728R*^, *PAX4*^*P287L*^, and *TP53*^*N247I*^ (Figure 1D). *TP53*^*N247I*^ was detected with a high cellular prevalence indicating loss of heterozygosity, which was supported by our DNA copy-number analysis. Additionally, we identified other putative truncal events, including deletion of *BRCA1*, *BRCA2*, and *PTEN* and amplification of *CCNE1* (Figure S1B), which are commonly altered in serous ovarian cancer (Bowtell et al., 2015, Patch et al., 2015). Among the private mutations we detected several potential driver mutations including *RUNX3*^*P246S*^ in the growing splenic lesion and *CSMD1*^*G1770R*^ in the primary tumor. Several private and shared branch mutations were found in different Rho GTPase-activating genes (*ARHGAP*), which inactivate Rho and Rac signaling involved in the control of cellular motility (Bernards and Settleman, 2004, Li et al., 2014).

### Transcriptomic Analysis Reveals Immune-Related Pathways Overexpressed in Regressing Tumors

**Figure 2:**
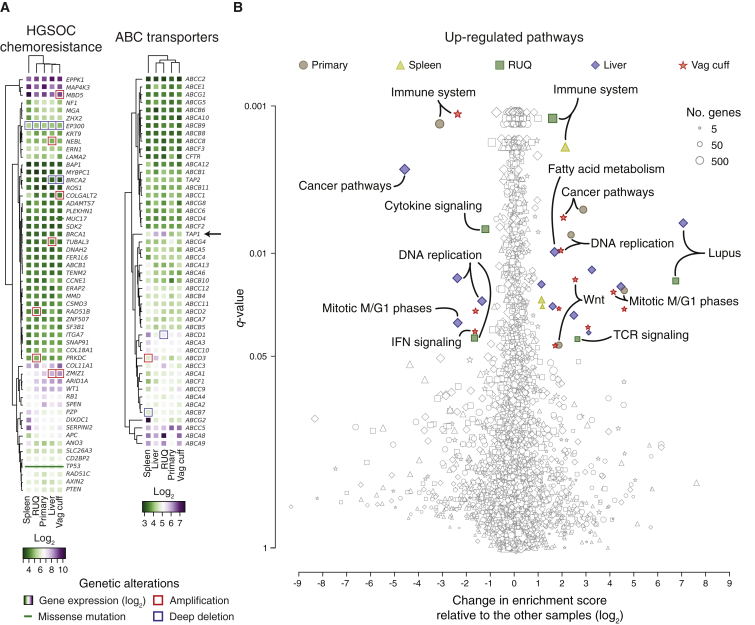
Differential Expression of Immune-Related Pathways in Heterogeneously Growing Tumors (A) Expression levels and genetic alterations of genes associated with chemotherapy resistance in HGSOC (Patch et al., 2015) and multidrug resistance. Amplification and deep deletion were defined as at least ± 2 median absolute deviations of copy-number alterations for each sample (Figure S1C). (B) Single-sample gene set enrichment analysis (Barbie et al., 2009, Subramanian et al., 2005) of upregulated pathways using the KEGG (Kanehisa and Goto, 2000, Kanehisa et al., 2016) and REACTOME (Fabregat et al., 2016) databases (Tables S2D and S2E). Significantly enriched pathways (q < 0.05) with at least ± 1 log_2_ change relative to the median of the other samples are colored (Table S2G). False-discovery rate adjusted p value (q value) was calculated using the Benjamini-Hochberg method.

**Figure S2:**
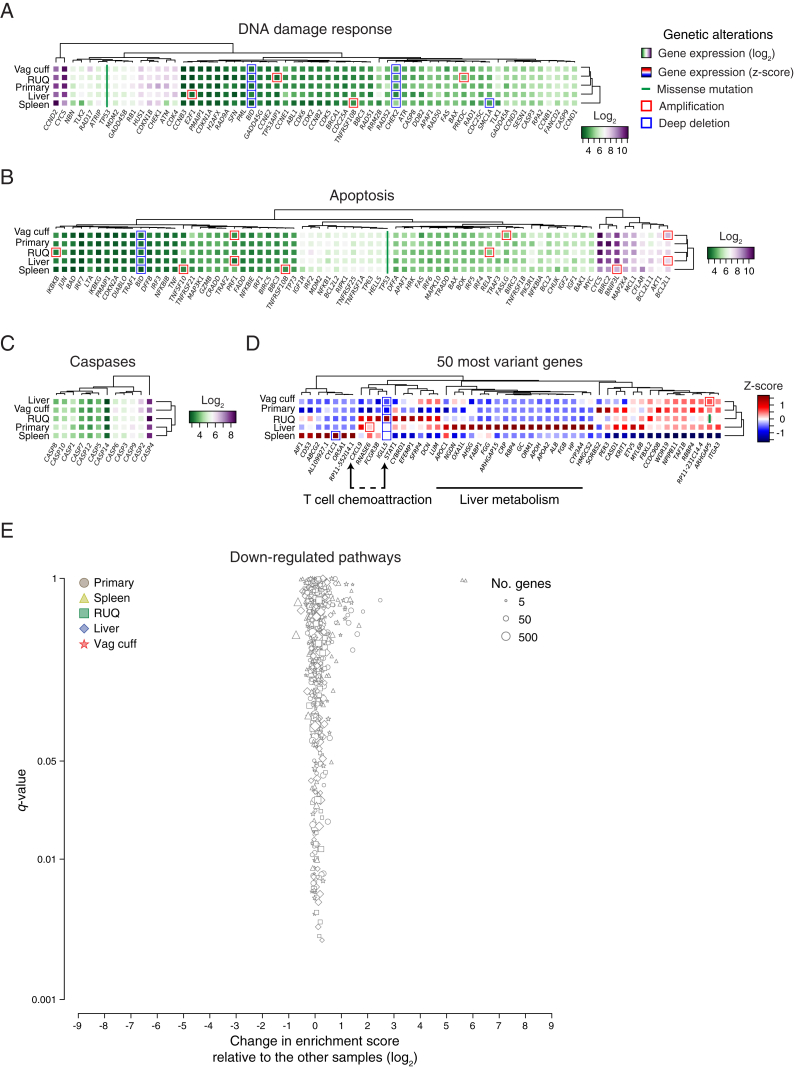
Gene Set Analysis of Transcript Abundance and Somatic Alteration Patterns across Samples, Related to Figure 2 and Table S2 (A–C) Gene-expression levels and genetic alterations of the DNA damage, apoptosis pathways, and caspases. (D) Expression levels of the 50 most variant genes according to their coefficient of variation (Table S2A). (E) Differential enrichment scores and enrichment q values of downregulated pathways between tumor samples (Tables S2D and S2E). No significantly enriched pathways (q < 0.05) with at least ± 1 log_2_ change relative to the median of the other samples were detected (Table S2G). False-discovery rate adjusted p value (q value) was calculated using the Benjamini-Hochberg method.

To evaluate whether genes involved in chemotherapy resistance were differentially altered between tumors and associated with regression and progression status, we analyzed somatic alterations and gene-expression data (Affymetrix transcript array) across the samples. After analyzing chemotherapy-resistance genes identified in HGSOC (Patch et al., 2015), as well as gene sets for multidrug resistance (ABC transporters), apoptosis, and DNA-damage response, we found no clear evidence of gene-expression or somatic-alteration patterns (mutations, DNA amplification, and deep deletion) that differed between progressing (primary, vaginal cuff, and spleen) and regressing/stable tumors (RUQ and liver) (Figures S2A–S2C). Interestingly, there was a trend that the ABC transporter *TAP1*, which is known for its function as a transporter of cytosolic peptides to the endoplasmic reticulum for HLA class I presentation (Bahram et al., 1991, Neefjes et al., 1993, Powis et al., 1991, Suh et al., 1994), was expressed at a higher level in the regressing tumors (Figure 2A).

To further identify potential differences between samples we analyzed gene sets and pathways in an unbiased manner with single-sample gene set enrichment analysis (ssGSEA) (Barbie et al., 2009, Subramanian et al., 2005). Using permutation-based false-discovery rate, we estimated the significance of the enrichment score for each pathway and performed an outlier analysis relating gene-set significance to the relative change in enrichment score between a given sample and the rest of the samples (Figure 2B**;** Tables S2D–S2G). The most significant and differentially enriched pathway found was the immune system pathway with a higher enrichment in the spleen and RUQ metastases and a lower enrichment in the primary and vaginal-cuff tumors (Table S2H). Further indicating immune activation, the systemic lupus erythematosus pathway was highly enriched in the RUQ and liver metastases (Table S2I), whereas TCR signaling pathways were preferentially enriched in the RUQ sample alone (Table S2J). Cancer and proliferation pathways, as well as Wnt signaling, were more enriched in the primary and vaginal-cuff tumors (Table S2K). No outlier gene sets were identified for the negatively enriched pathways (Figure S2E; Tables S2D–S2F).

To investigate the gene-expression differences between the samples on an unbiased individual gene level, we calculated the coefficient of variation of the expression levels for each gene across samples (Table S2A). We found that among the most variably expressed genes, besides lipid metabolic process-related genes in the liver, the T cell chemo-attractant *CXCL9* was predominantly expressed in the RUQ and liver metastases, as well as *STAT1*, which has been implicated in the regulation of *CXCL9* expression (Liao et al., 1995, Satoh and Tabunoki, 2013) (Figure S2D). No relevant mutations in immune-related molecules were identified except for truncal mutations in the MHC class I polypeptide-related sequence B (*MICB*) (Table S1A), which is a stress-induced ligand recognized by NKG2D receptors on CD8αβ and γδ T cells, as well as NK cells (Bauer et al., 1999, Groh et al., 1999).

### Heterogeneous Immune Cell Infiltration in Growing and Regressing Lesions

**Figure 3:**
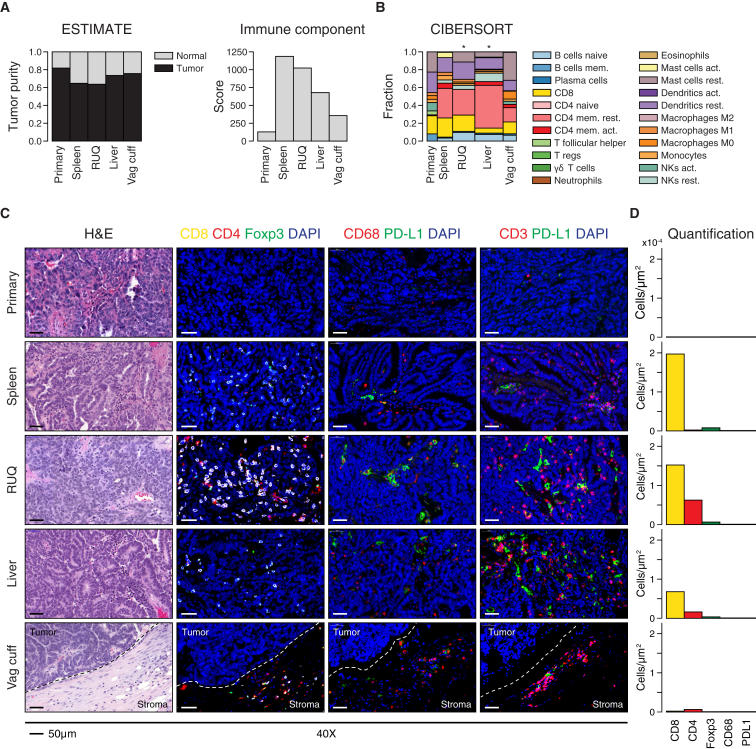
Immune Infiltration Status Shows Heterogeneous Microenvironments across Tumor Samples (A) Tumor purity and immune component estimated by analyzing Affymetrix-based transcriptomics (Table S3A) (Yoshihara et al., 2013). (B) Fractions of immune cell subsets in tumor samples inferred from gene-expression data using CIBERSORT (Newman et al., 2015). Width of bars is proportional to the −log_10_ p value of the deconvolution (Table S3B). CIBERSORT empirical p value, ^∗^p < 0.05. (C) Representative images of hematoxylin and eosin staining of tumor samples and immunofluorescence staining for DAPI, cytotoxic T cells (CD8^+^), helper T cells (CD4^+^FOXP3^−^), T cells (CD3^+^), T-regs (CD4^+^FOXP3^+^), macrophages (CD68^+^), and immune-checkpoint PD-L1. Complete slides are shown in Figure S3. (D) Image-based cell quantification of whole slides (Table S3C).

To investigate the immune infiltration status of the tumors, we used ESTIMATE to analyze tumor purity and overall stromal and immune components (Yoshihara et al., 2013). The lowest tumor purities and highest immune infiltration scores were found in the RUQ, liver, and spleen samples (Figure 3A). Furthermore, we deconvolved the gene-expression data using CIBERSORT (Newman et al., 2015) as a first approach to dissect infiltration of specific immune cell subsets in the tumors. We found that the largest immune cell components corresponded to CD8^+^ and CD4^+^ T cells in RUQ, liver, and spleen tumors, although the overall CIBERSORT deconvolution p value was only significant for RUQ and liver tumors (Figure 3B). In contrast, the primary and vaginal-cuff tumors had low immune cell ESTIMATE scores and insufficient levels of immune cell transcripts to confidently apply CIBERSORT (Tables S3A and S3B), together suggesting a low or absent immune component present in these tumors.

**Figure S3:**
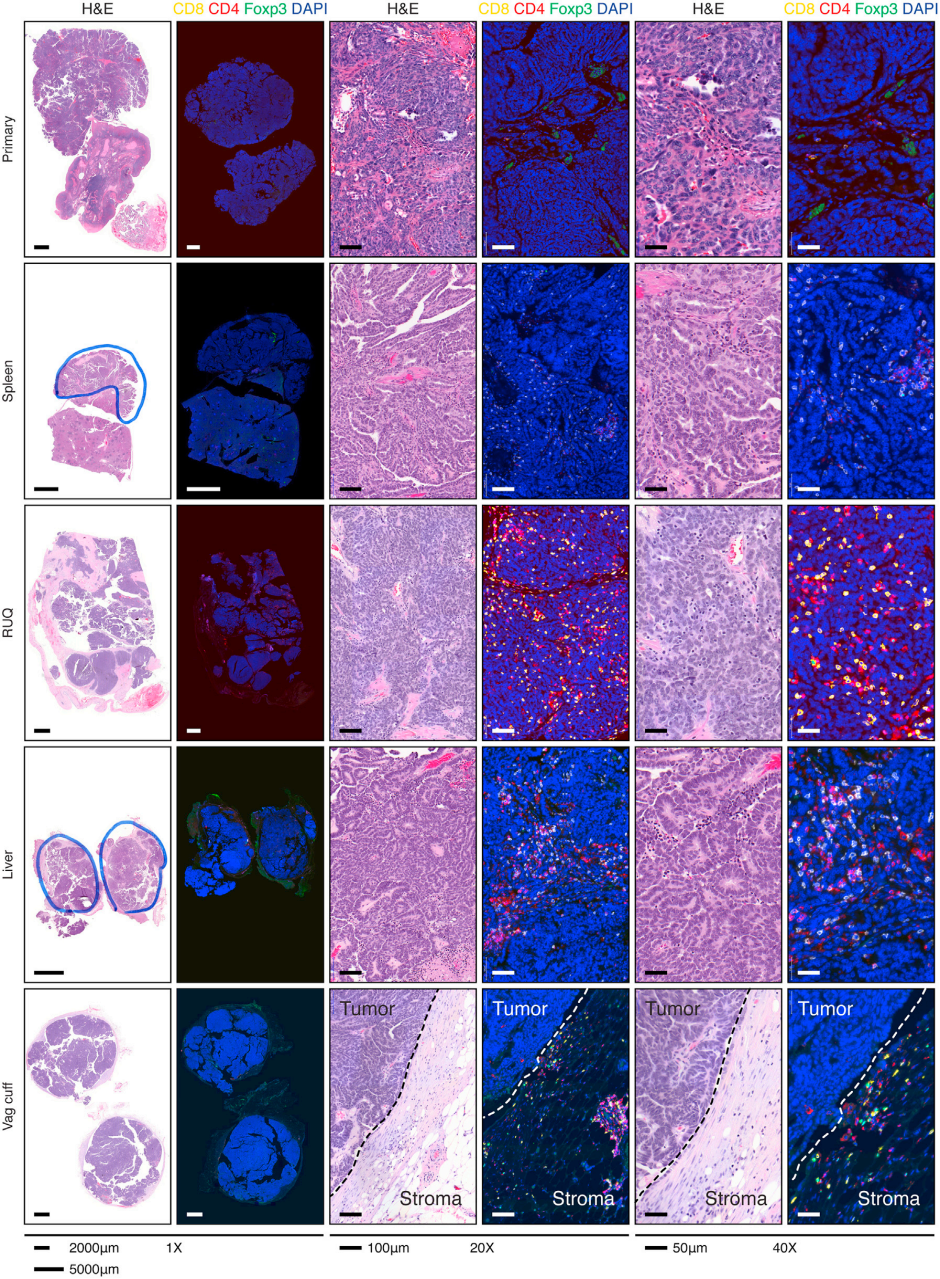
Complete Slide Hematoxylin and Eosin and Immunofluorescent Staining, Related to Figure 3 and Table S3 Hematoxylin and eosin staining of tumor samples. Immunofluorescence staining for cytotoxic T cells (CD8^+^), helper T cells (CD4^+^FOXP3^−^), and regulatory T cells (CD4^+^FOXP3^+^).

Following this analysis, samples were immuno-fluorescently co-stained for T cell markers CD4, CD8, and the T regulatory cell marker FOXP3, double stained for PD-L1 and macrophage marker CD68, as well as double stained for PD-L1 and the T cell marker CD3 (Figures 3C and S3A). Consistent with the transcriptomic deconvolution analyses, the primary tumor demonstrated no T cell infiltration and was negative for PD-L1 and CD68 (Figures 3C, 3D, and S3A; Table S3C). The vaginal-cuff lesion, which was growing at the time of surgical resection, did display a T cell population; however, these cells bordered but did not infiltrate the tumor. The splenic lesion, which was also progressing at the time of resection, albeit at a much more modest rate than the vaginal-cuff lesion, demonstrated a CD8^+^ infiltrate. Finally, the RUQ and liver metastases, which were regressing and stable, respectively, at the time of surgical resection, displayed a strong CD4^+^ and CD8^+^ infiltrate. In summary, the transcript and IF analyses suggested that each tumor site displayed a unique tumor-immune microenvironment ranging from immune cell inclusion to exclusion.

### Regressing Metastases Show T Cell Oligoclonal Expansion

**Figure 4:**
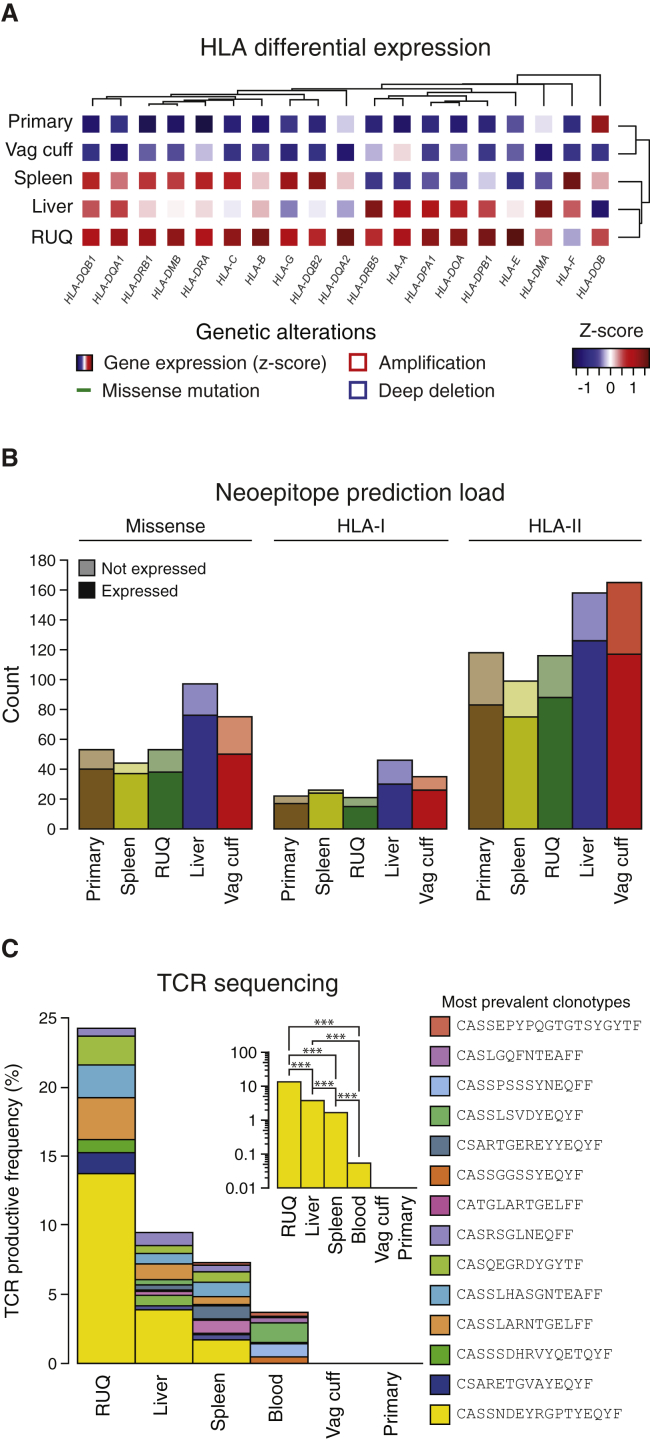
Higher *HLA* Expression and T Cell Oligoclonal Expansion Detected in Regressing Tumors (A) *HLA-I* and *II* gene differential expression across samples (Table S2A). (B) Number of predicted neoepitopes per sample (Tables S4B–S4D). (C) TCR sequencing of FFPE tumor samples and blood. The most prevalent TCR clonotypes (top 5 for each sample and blood) are shown (Table S5A). The blood sample was collected from the patient 550 days after secondary debulking (Figure S6A). Inset shows detection of the most frequent TCR rearrangement (CASSNDEYRGPTYEQYF) and its abundance comparison between samples (two-sided binomial tests with Benjamini-Hochberg multiple test correction, ^∗∗∗^ q < 0.001).

It is known that genetic alterations in HLA-I molecules are associated with escape of cancer cells from CD8^+^ T cell recognition (Shukla et al., 2015). The patient’s *HLA* alleles were determined experimentally by conventional PCR-based *HLA* typing and computationally on exome data by OptiType (Szolek et al., 2014) and POLYSOLVER (Shukla et al., 2015) independently, yielding the same results (Table S4A). We searched copy-number alterations as well as mutations by applying POLYSOLVER, a specific computational pipeline for *HLA-I* typing and mutation detection in the *HLA-I* genes; however, no genetic alterations were detected. We then assessed gene expression and found that all *HLA-I* genes were expressed in the tumors (Tables S2A–S2C); however, compared to primary and vaginal-cuff samples, an overall higher expression of *HLA* genes was observed in the RUQ and liver samples, with a lesser extent seen in the spleen sample (Figure 4A).

**Figure S4:**
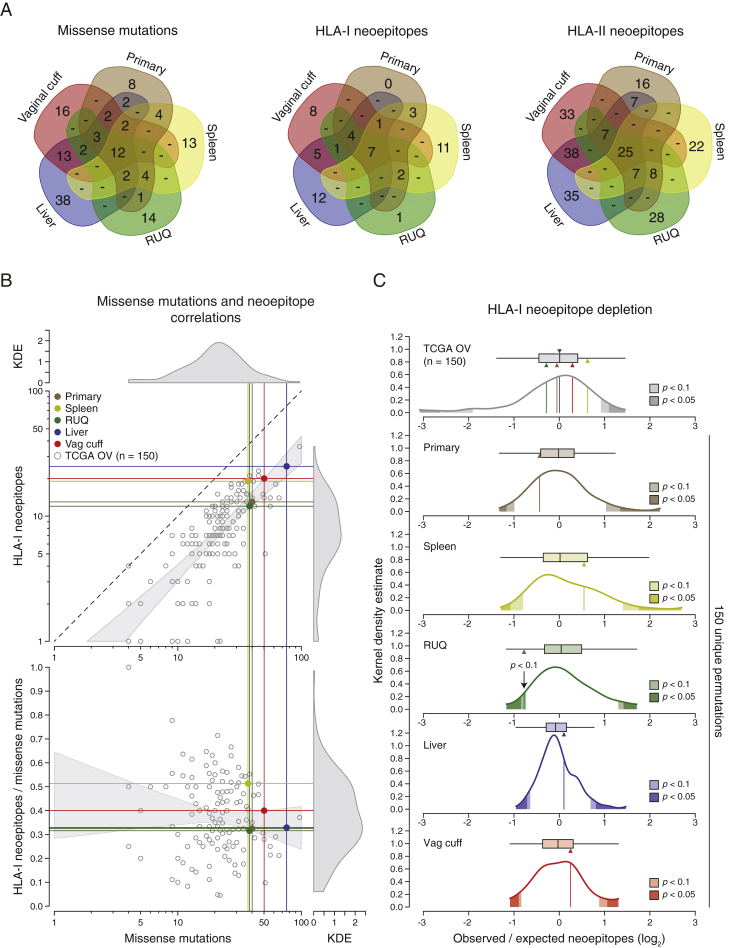
Neoepitope Distributions and HLA-I Neoepitope Depletion Analysis, Related to Figure 4 and Table S4 (A) Number of unique and overlapping expressed missense mutations, HLA-I and II neoepitopes between samples (Table S4D). (B) Correlations between expressed missense mutations and predicted HLA-I neoepitopes using NetMHC applied to TCGA ovarian samples (n = 150) and the primary and metastatic tumors (Tables S4E–S4G). KDE = kernel density estimate. (C) Top: Estimated neoepitope deviation from expected in the five tumor samples compared to TCGA ovarian cancer samples (n = 150). The expected number of neoepitopes was calculated by taking into account the expected number of missense mutations and the number of silent mutations according to Rooney et al., 2015 (see STAR Methods). Bottom: Neoepitope depletion analysis of 150 random unique permutations of the patient’s tumors (primary, spleen, RUQ, liver, and vaginal cuff) and their mutations. Each sample was compared against its own 150 unique permutations to control for the number of mutations (Tables S4H and S4I). Two-sided empirical p values were calculated from each distribution.

We next estimated the neoepitope landscape of the samples by mapping missense mutations to their amino acid sequences, in silico generating the mutant peptide sequences, and predicting the mutant peptide-HLA binding affinities to the patient’s HLAs. The predictions were performed using the NetMHC algorithm with HLA specific cut-offs for HLA-I (Lundegaard et al., 2008, Nielsen et al., 2003, Paul et al., 2013) and consensus scores for HLA-II (Kim et al., 2012, Kreiter et al., 2015). The tumors with the highest mutation and neoepitope loads for both HLA class I and HLA class II were the liver and vaginal cuff, which also had the highest number of missense mutations (Figure 4B). We also investigated whether there were shared neoepitopes or mutations present in the RUQ (regressing) and liver (stable) metastases alone, i.e., not present in the other tumors. No shared mutations between RUQ and liver alone were detected (Figure S4A); therefore, it did not appear that a shared neoepitope or mutation alone explained the behavior of the non-progressing tumor sites.

**Figure S5:**
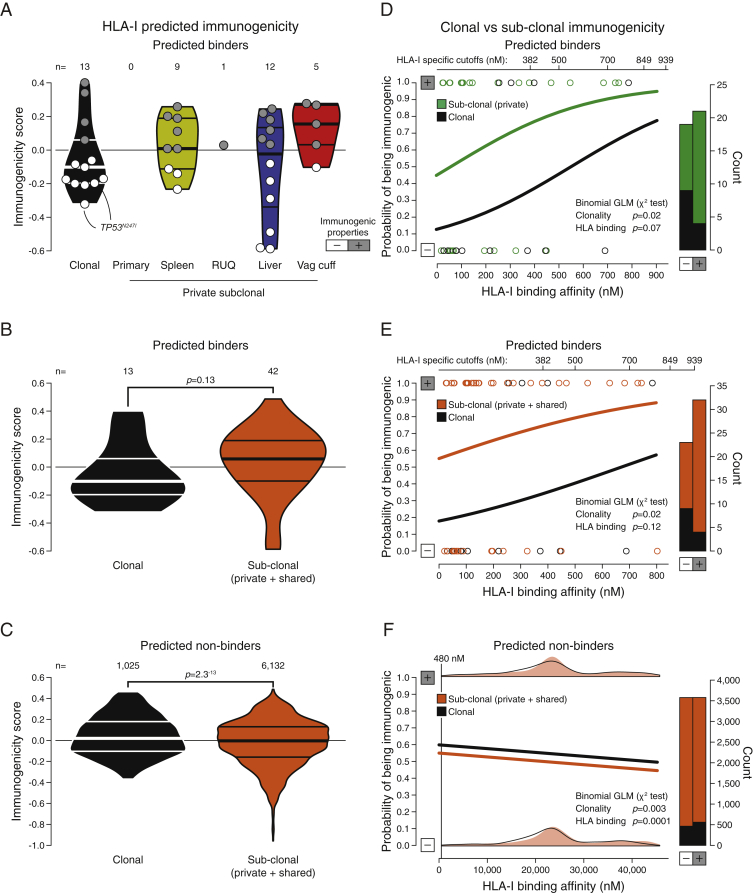
Predicted Immunogenicity of HLA Class I Neoepitopes, Related to Figures 1 and 4 and Tables S1 and S4 (A) Predicted immunogenic properties of trunk (clonal) and private HLA-I neoepitopes. Positive immunogenicity scores have biochemical properties associated with higher immunogenicity that outweigh properties associated with lower immunogenicity, and vice versa for negative scores (Calis et al., 2013). Horizontal lines within violin plots show the median and interquartile range of the data distribution. (B and C) Comparison between clonal and sub-clonal (including shared between two or more samples but not all) predicted immunogenicity of predicted binders and non-binders (two-sided Mann-Whitney rank test). Horizontal lines within violin plots show the median and interquartile range of the data distribution. (D–F) Probability of an HLA-I neoepitope having immunogenic properties considering its clonality and HLA-I binding affinity using the neoepitope data in (A), (B), and (C), respectively. Clonal neoepitopes have a lower probability of having immunogenic properties than sub-clonal predicted binders (chi-square test, p = 0.02). For non-binders (NetMHC score > HLA-I specific cutoff), clonal neoepitopes have a higher probability of having immunogenic properties (chi-square test, p = 0.003), as well as peptides with higher HLA-I affinities (chi-square test, p = 0.0001), although the absolute differences are minor. No significant interaction between clonality and predicted HLA-I binding affinity was detected for either binders or non-binders. GLM = generalized linear model.

As an active CD8 T cell infiltration can exert a selective pressure at the neoepitope level (DuPage et al., 2012, Matsushita et al., 2012, Teng et al., 2015, Tran et al., 2016, Verdegaal et al., 2016), we further interrogated the neoepitope landscape by analyzing potential evidence of neoepitope depletion using an approach adopted from a report analyzing TCGA data (Rooney et al., 2015). Relative to the other samples from the patient, the regressing RUQ tumor showed a consistent—yet non-significant—tendency of neoepitope depletion (p < 0.1 by two-sided empirical p value; Figures S4B and S4C). This result is in line with a recent report showing neoepitope depletion in tumors with higher levels of immune signatures in colorectal cancer (Davoli et al., 2017). We then predicted the intrinsic immunogenicity of neoepitopes by analyzing the biochemical properties of peptides that are predicted to be associated with T cell-epitope recognition (Calis et al., 2013). We observed that there was a significant effect of neoepitope clonality on the probability of a neoepitope having immunogenic properties, with clonal neoepitopes being predicted as less immunogenic (p = 0.02 by chi-square test; Figures S5A–S5D). Using the predicted non-binders instead of binders in a control analysis, the opposite trend was observed as there was a small but significant effect of clonal mutations being predicted as more immunogenic (p = 0.003 by chi-square test; Figure S5F). Although preliminary, these analyses indicate a potential negative selection process at the neoepitope level.

**Figure S6:**
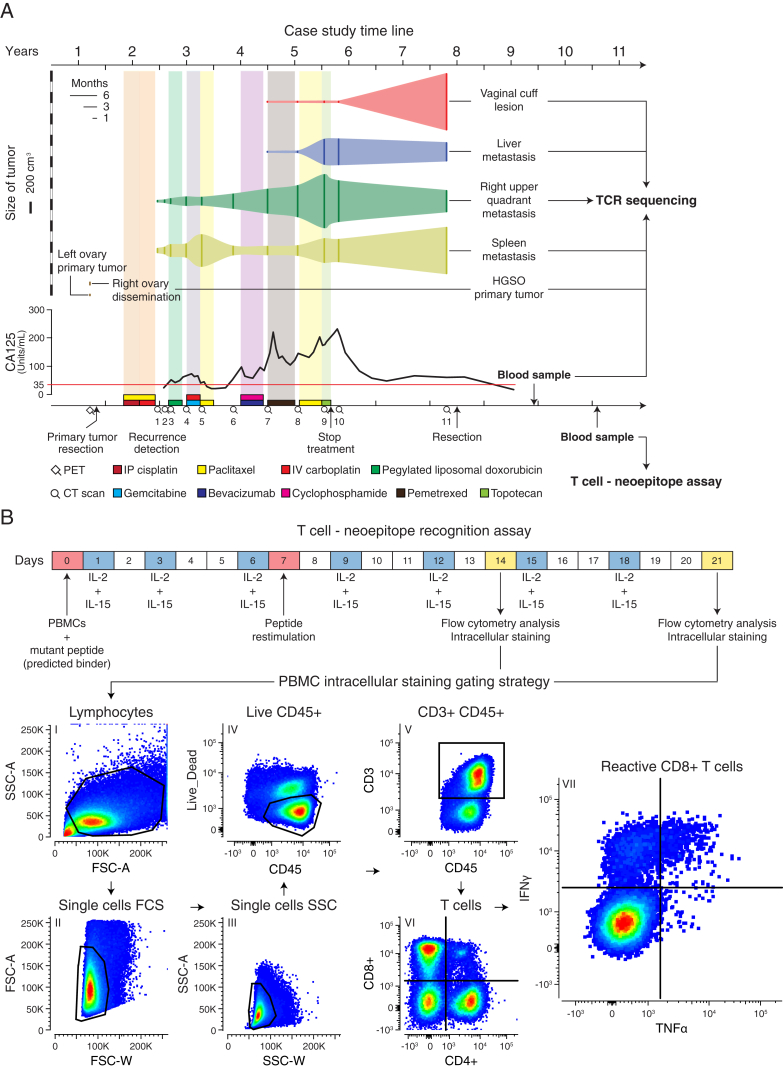
PBMCs Sample Timeline and T Cell-Neoepitope Recognition Assay, Related to Figures 4 and 5 and Table S5 (A) Blood samples obtained from the patient 550 and 978 days after resection were used for TCR sequencing and T cell – neoepitope recognition assays respectively. (B) Experimental setup and flow cytometry gating strategy for the T cell –neoepitope recognition assays (intracellular cytokine staining assay) with surface staining of CD3, CD4, CD8, CD45, and intracellular staining of IL-4, IFN-γ, TNF-α. PBMC = peripheral blood mononuclear cells.

To evaluate a T cell response in the tumors, we investigated whether T cell clonal expansion could be detected in the tumor samples. To this end, we performed in situ TCR sequencing on each sample and on peripheral blood from the patient sampled 550 days after tumor resection (Figures 4C and S6A; Table S5A). We detected a T cell expansion in the RUQ metastasis with a dominant clone accounting for 13% of all productive T cell receptors sequenced. The expanded clone was also detected in the liver and spleen metastases and strikingly also in the blood of the patient. Though, the clonal frequency in the RUQ metastasis was significantly higher than that in the other samples (q < 0.001 by two-sided binomial tests with BH correction). In contrast, no T cell receptors were detected in the primary and vaginal-cuff tumors, further supporting their lack of T cell infiltrate.

### Peripheral Blood CD8^+^ T Cells React against Predicted Neoepitopes

**Figure 5:**
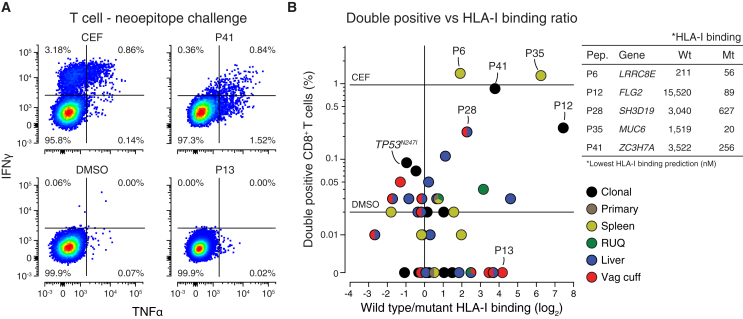
Predicted Neoepitopes with Higher Mutant than Wild-Type HLA-I Binding Affinity Elicit a T Cell Response (A) Representative scatterplots of TNF-α and IFN-γ intracellular cytokine staining of CD8^+^ T cells after 21 days of culture with CEF peptides or DMSO as positive and negative controls or the predicted mutant peptides (Figure S6B). CEF = Cytomegalovirus, Epstein-Barr virus, Influenza virus. (B) Percentage of CD8^+^ T cells with double-positive intracellular staining (TNF-α and IFN-γ) after incubation with each of the 43 predicted HLA-I neoepitopes, and HLA-I predicted binding affinity wild-type to mutant ratio (Table S5B). Mutation in gene *FLG2*^*E1608K*^ (P12) was found to be clonal after manual inspection in IGV (Table S1A).

Since expanded T cell clones detected in the tumors were still detected in the patient’s blood sampled 1 year 6 months (550 days) after resection, we decided to test whether circulating T cells could react against any of the predicted neoepitopes. We sampled blood from the patient again, this time at 2 years 8 months (978 days) after resection, and isolated peripheral blood mononuclear cells (PBMCs) (Figure S6A). We performed an ICS assay lasting 21 days, where PBMCs were cultured with each of the mutant peptides (n = 43) predicted to have at least one HLA-I neoepitope, as a mutant peptide (17-mer) can have more than one predicted binder (9-mer) (Figure S6B). Importantly, the likelihood of observing T cell reactivity by the ICS assays is low due to the low frequency of T cell precursors in the blood and the limited representation of the total TCR repertoire in each peptide challenge experiment (5 × 10^5^ cells per well) (Rizvi et al., 2015). Despite the high false-negative rate generally observed with the ICS assay, we found CD8^+^ T cells reactive against several mutant peptides showing cytokine activation levels (IFN-γ and TNF-α) similar to the positive control consisting of a mixture of viral-derived epitopes (Figures 5A and 5B; Table S5A). Of the top five reactive peptides detected, all had a higher mutant to wild-type predicted HLA-I binding affinity (inset, Figure 5B). With limited material available, we focused on the top hits and repeated the ICS experiment and again found reactivity with peptide 12, which was derived from a clonal mutation in *FLG2*^*E1608K*^ (Table S1A), and peptide 6, which was derived from a private mutation in *LRRC8E*^*C629Y*^ in the splenic tumor.

## Discussion

**Figure S7:**
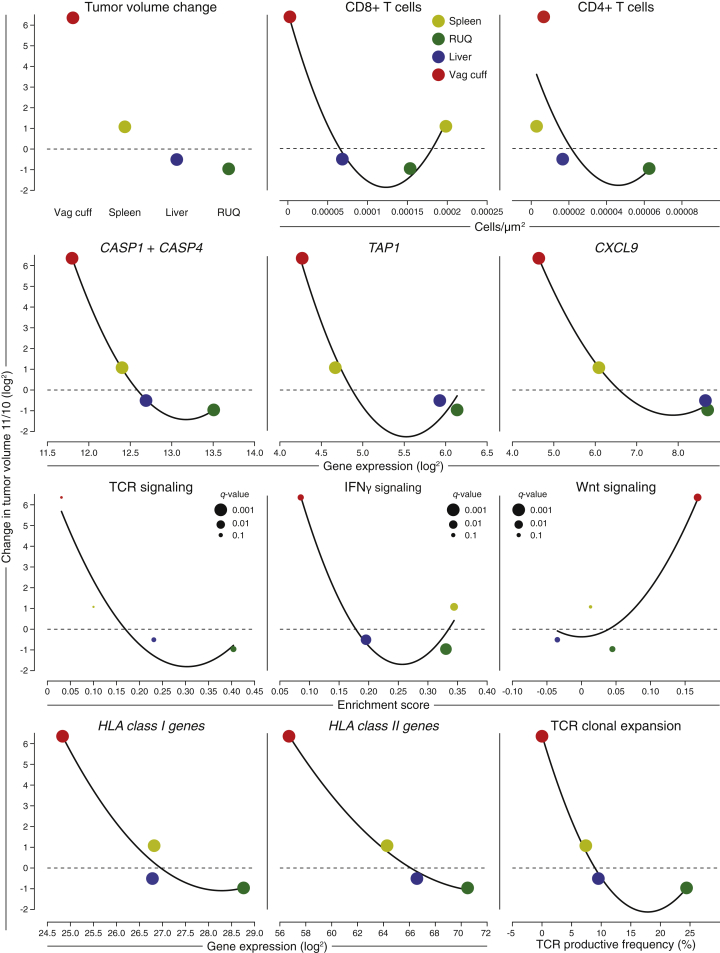
Overall Associations between Tumor Fates and Tumor-Immune Microenvironmental Features, Related to Figures 1, 2, 3, and 4 and Tables S1, S2, S3, and S4 Cellular and molecular associations with change in tumor growth. Change in tumor growth (y axis) was calculated by dividing the tumor volume at CT scan 11 by the tumor volume at CT scan 10 (Figure 1B). Fitted curves are 2^nd^ order polynomial regression lines plotted for trend visualization rather than prediction purposes. *Capase 1* and *4* are considered inflammatory caspases involved in a type of apoptosis related to immune response called pyroptosis. The enrichment score x axis and the *q*-values come from the ssGSEA analysis. *HLA-I* genes include *HLA-A*, *B*, *C*, *E*, and *F*. HLA-II genes include *HLA-DPA1*, *DMA*, *DRA*, *DQA1*, *DMB*, *DPB1*, *DQB2*, *DRB5*, *DRB1*, *DQB1*, and *DOA*.

The natural history of ovarian cancer typically features remissions of decreasing length, leading to premature death (Bowtell et al., 2015). In this unusual case, the divergent fates of the tumors show an overall association with multiple molecular and cellular features at the tumor-immune interface (Figure S7). For example, the shrinking RUQ tumor was heavily infiltrated with CD4 and CD8 T cells and had evidence of active CD8 T cell surveillance with expansion of specific TCR clonotypes. The stable liver tumor also exhibited immune infiltration, but at a lower level and with fewer expanding T cell clones. The spleen tumor was growing modestly at the time of resection and presented with intermediate tumor-immune microenvironment features. Finally, the growing vaginal-cuff and the primary tumor exhibited complete immune cell exclusion. The TCR clone most prevalent in the non-progressing tumors was also detected in the blood of the patient 18 months after the metastases were resected, and clonal neoepitopes induced a CD8^+^ T cell response from PBMCs obtained nearly 3 years after surgery. The two most extreme tumors, the RUQ and the vaginal cuff, had a consistently divergent pattern of molecular features associated with immune activation (*HLA* expression, *IFN-γ*, *CXCL9*, *TAP1*, etc.) and immune suppression (Wnt signaling). Importantly, the observed features that relate to progression/regression status are correlative and do not per se prove any bona fide mechanism nor negate the fact that chemotherapy could have influenced the divergent fates. In sum, we find evidence of distinct tumor-immune microenvironments among differentially growing metastases within the same individual.

Particular findings of this study may have important clinical implications if they are corroborated in large cohorts. In this patient with advanced HGSOC we observed distinct tumor-immune microenvironments in the five sampled tumors (primary and four recurrent tumors). The mutation and predicted neoepitope space alone did not explain the different regressing/progressing behavior of the metastatic samples. In contrast to recent studies of resistance to immunotherapy, no mutations were detected in the antigen presentation machinery (Giannakis et al., 2016, Rooney et al., 2015, Shukla et al., 2015), *B*_*2*_*-microglobulin* (Challa-Malladi et al., 2011, Rooney et al., 2015, Zaretsky et al., 2016), the IFN-γ pathway (Benci et al., 2016, Zaretsky et al., 2016), or *HLA-I* genes (Rooney et al., 2015, Shukla et al., 2015, Tran et al., 2016) in the growing tumors. Instead of specific neoepitopes present in regressing samples, T cell reactivity against clonal neoepitopes was detected. Interestingly, all neoepitopes that elicited a CD8^+^ T cell response had higher mutant to wild-type HLA-I predicted binding affinity. The lack of tumor-specific somatic alterations in the regressing and stable tumors alone puts forward the idea that non-somatic factors in the tumor microenvironment may have been playing a critical role in the immune response and overall fate of the tumors. For example, *STAT1* and *CXCL9* were highly expressed in the RUQ and liver metastases; CXCL9 is well known as a potent T cell chemokine (Liao et al., 1995, Rainczuk et al., 2012), and high expression of *CXCL9* and *CXCL10* correlate with enhanced T cell infiltration of tumors and better survival of ovarian cancer patients (Bronger et al., 2016). In contrast, the vaginal cuff growing metastasis had a higher enrichment score in the Wnt pathway, which has been implicated as a mechanism that impairs recruitment of dendritic cells and prevents T cell infiltration in autochthonous mouse melanoma models via a *CXCL9*- and *CXCL10*-dependent mechanism (Spranger et al., 2015, Spranger et al., 2017). Although a direct link between tumor fate and the observations found in this patient cannot be proven with the available samples, this case emphasizes the importance of an integrative approach to understand the molecular mechanisms governing the interaction between the tumor and its immune microenvironment (Miao and Van Allen, 2016).

As in any case study, the present study has notable limitations. It involves only one patient, and thus further studies are needed to determine whether the principles discovered here apply to other patients. Furthermore, the interplay between treatment, somatic mutations, the immune system, and heterogeneous fates of the tumors cannot be untangled in this clinical case. For example, it is feasible that the multiple chemotherapy interventions for this patient contributed to shaping the somatic mutations and the microenvironment of the tumors, but due to the availability of samples and descriptive nature of the study this could not be explored further. Despite such limitations, this case provides evidence for differential tumor-immune responses existing in metastases of the same individual, related not only to genetic alterations but also to the tumor-immune microenvironment, which to our knowledge has not yet been demonstrated in patients with ovarian cancer. Also, most studies on the tumor-immune microenvironment have been conducted in primary tumors (Teng et al., 2015), with the exception of a study of matched primary and metastatic tumors, which concluded that the immune contexture globally recapitulates that of the primary (Remark et al., 2013). In contrast, the case of recurrent HGSOC presented here clearly shows the opposite: that tumor-immune microenvironments, between primary tumor and metastases, and between metastases, can be heterogeneous within a patient.

Previous genomic and immune profiling of multiple lesions in patients have also shed light on tumor heterogeneity and its implications on tumor evolution (Gerlinger et al., 2012), disease progression (Ascierto et al., 2017), and immune control (McGranahan et al., 2016, Şenbabaoğlu et al., 2016, Sridharan et al., 2016). For example, tumors that are genetically more heterogeneous have less immune infiltrates (Şenbabaoğlu et al., 2016) and less benefit from checkpoint-blockade immunotherapies (McGranahan et al., 2016). It has been shown that T cell infiltration and gene expression of immune-related genes correlate with response to checkpoint-blockade immunotherapy in melanoma (Chen et al., 2016). Additionally, analyses of synchronous resected metastases with differential progression in patients with melanoma has shown that intra-patient metastases present not only genetic heterogeneity but also immune-infiltration heterogeneity of immune cell types and T cell clonality between samples (Reuben et al., 2017). A rapid autopsy study of a patient with metastatic melanoma treated with anti-PD-1 therapy showed that resistant metastases overexpressed genes related to extracellular matrix and neutrophil function (Ascierto et al., 2017). Interestingly, association between Wnt signaling and lack of T cell infiltration was also observed in a patient with adenoid cystic carcinoma where serial biopsies from the same patient were analyzed, and different expression profiles between primary and metastatic deposits were also detected (Sridharan et al., 2016). Finally, a plethora of molecular mechanisms and types of cells influencing the tumor-immune microenvironment have been described in different tumor types, leading to important advances in immunotherapy (Joyce and Fearon, 2015, Melero et al., 2014, Sharma et al., 2017). Unfortunately, the promise of immunotherapy has not been as successful in ovarian cancer as it has been in other tumor types (Homicsko et al., 2016) despite the fact that it was recognized more than a decade ago that T cell infiltration is a key element for patient outcome in this disease (Zhang et al., 2003). We believe that the growing evidence of differential genomic, transcriptomic, and immune profiles between and within patients will eventually provide new key elements to target in ovarian cancer and other tumor types. However, this task will require extensive and deep systematic analyses along with longitudinal data, as the differences between metastases and coexistence of tumor-immune microenvironments within a patient are likely to be dynamic and sensitive to intrinsic (e.g., mutations and cell-cell communication) and extrinsic perturbations (e.g., prior treatment and microbiome; Sivan et al., 2015, Vetizou et al., 2015).

In conclusion, this case study provides evidence of divergent tumor genetics, tumor microenvironments, and immune activation within a single patient with advanced ovarian cancer. If this phenomenon proves generalizable, then the inter-site heterogeneity described here bespeaks a profound clinical challenge for the use of cytotoxic, targeted, and immuno-therapies. This observation, although made in an exceptional long-term survivor patient, may explain the frequent heterogeneous responses seen clinically but insufficiently documented by the limited radiographic measurements provided by the Response Evaluation Criteria in Solid Tumors (RECIST). Given the data presented in this study, it will be essential to understand not only how to therapeutically target genomic heterogeneity between and within metastases but also how to successfully mobilize an anti-tumor immune response able to control all metastases in advanced cancers.

## STAR★Methods

### Key Resources Table

**Table:** 

REAGENT or RESOURCE	SOURCE	IDENTIFIER
**Antibodies**		

Rabbit Anti-CD4 Monoclonal Antibody	Ventana	Cat# 790-4423; RRID: AB_2335982
Goat Anti-rabbit IgG Antibody	Vector Laboratories	Cat# PK6101; RRID: AB_2336820
Mouse Anti-FoxP3 Monoclonal Antibody	abcam	Cat# ab20034; RRID: AB_445284
Biotinylated Horse Anti-mouse IgG	Vector Laboratories	Cat# MKB-22258; RRID: AB_2336180
Rabbit Anti-CD8 Monoclonal Antibody	Ventana	Cat# 790-4460; RRID: AB_2335985
Rabbit Anti-PD-L1 Monoclonal Antibody	Cell Signaling	Cat# 13684
Mouse Anti-CD68 Monoclonal Antibody	Dako	Cat# M0814; RRID: AB_2314148
Rabbit Anti-CD3 Polyclonal Antibody	Dako	Cat# A0452; RRID: AB_2335677
Mouse Anti-CD8 Monoclonal Antibody, Phycoerythrin Conjugated, Clone SK1	BD Biosciences	Cat# 340046; RRID: AB_400005
CD4 Monoclonal Antibody (OKT4 (OKT-4)), PerCP-Cyanine5.5, eBioscience	Thermo Fisher Scientific	Cat# 45-0048-42; RRID: AB_10804390
Mouse Anti-CD3 Monoclonal Antibody, Pacific Blue Conjugated, Clone UCHT1	BD Biosciences	Cat# 558117; RRID: AB_397038
Mouse Anti-Human CD45 Monoclonal Antibody, APC-H7 Conjugated	BD Biosciences	Cat# 560178; RRID: AB_1645479
LIVE/DEAD Fixable Aqua Dead Cell Stain	Thermo Fisher Scientific	L34957
TNF-α Monoclonal Antibody (MAb11), PE-Cyanine7, eBioscience	Thermo Fisher Scientific	Cat# 25-7349-82; RRID: AB_469686
IFN-γ Monoclonal Antibody (GZ-4), FITC, eBioscience	Thermo Fisher Scientific	Cat# BMS107FI; RRID: AB_10596520

**Biological Samples**		

Primary High Grade Serous Ovarian Cancer	MSKCC	N/A
Spleen metastasis	MSKCC	N/A
Right Upper Quadrant metastasis	MSKCC	N/A
Liver metastasis	MSKCC	N/A
Vaginal Cuff metastasis	MSKCC	N/A
Blood samples	MSKCC	N/A

**Chemicals, Peptides, and Recombinant Proteins**		

Fixation/Permeabilization Solution Kit	BD Biosciences	Cat# 554714
17-mer custom peptides (n = 43)	GenScript	Cat# SC1487
CEF peptide pool “classic”	C.T.L.	Cat# CTL-CEF-001
Recombinant human IL-2, Proleukin	Chiron	N/A
Recombinant human IL-15	Peprotech	Cat# 200-15

**Deposited Data**		

Raw whole-exome sequencing primary sample	This paper	BioSample: SAMN06199513
Raw whole-exome sequencing primary sample	This paper	BioSample: SAMN06199514
Raw whole-exome sequencing primary sample	This paper	BioSample: SAMN06199515
Raw whole-exome sequencing primary sample	This paper	BioSample: SAMN06199516
Raw whole-exome sequencing primary sample	This paper	BioSample: SAMN06199517
Raw whole-exome sequencing primary sample	This paper	BioSample: SAMN06199518
Raw microarray data (all samples)	This paper	GEO: GSE92780

**Software and Algorithms**		

MuTect v1.1.4	Broad Institute	http://archive.broadinstitute.org/cancer/cga/mutect
Integrative Genomics Viewer v2.3.61	Broad Institute	http://software.broadinstitute.org/software/igv/
Phangorn v2.0.2	CRAN	https://cran.r-project.org/web/packages/phangorn/index.html
CopywriteR v2.2.0	Bioconductor	http://bioconductor.org/packages/release/bioc/html/CopywriteR.html
ABSOLUTE v1.0.6	Broad Institute	http://archive.broadinstitute.org/cancer/cga/absolute
PyClone v2.7.11	Shah Lab	http://compbio.bccrc.ca/software/pyclone/
GSVA v1.24.1	Bioconductor	https://bioconductor.org/packages/release/bioc/html/GSVA.html
Affymetrix Expression Console Software	Affymetrix	https://www.thermofisher.com/uk/en/home/life-science/microarray-analysis/microarray-analysis-instruments-software-services/microarray-analysis-software/affymetrix-expression-console-software.html
Affymetrix Transcriptome Analysis Console	Affymetrix	https://www.thermofisher.com/uk/en/home/life-science/microarray-analysis/microarray-analysis-instruments-software-services/microarray-analysis-software/affymetrix-transcriptome-analysis-console-software.html
ESTIMATE v1.0.13	MD Anderson Bioinformatics	http://bioinformatics.mdanderson.org/main/ESTIMATE:Overview
CIBERSORT Jar v1.05	Stanford University	https://cibersort.stanford.edu/
OptiType v1.0	GitHub	https://github.com/FRED-2/OptiType
POLYSOLVER	Broad Institute	http://archive.broadinstitute.org/cancer/cga/polysolver
Samtools v0.1.19	SAMtools	http://samtools.sourceforge.net/
Novocraft v3.02.05	Novocraft	http://www.novocraft.com/
MuTect	Broad Institute	http://archive.broadinstitute.org/cancer/cga/mutect
NetMHC v3.4	Immune Epitope Database and Analysis Resource	http://tools.iedb.org/mhci/download/
NetMHC II v2.2	Immune Epitope Database and Analysis Resource	http://tools.iedb.org/mhcii/download/
Sturniolo	Immune Epitope Database and Analysis Resource	http://tools.iedb.org/mhcii/download/
Immunogenicity	Immune Epitope Database and Analysis Resource	http://tools.iedb.org/immunogenicity/download/
immunoSEQ ANALYZER v3.0	Adaptive biotechnologies	http://www.adaptivebiotech.com/immunoseq/analyzer
FlowJo v10.3	FlowJo, LLC	https://www.flowjo.com/solutions/flowjo
BD FACSDiva v8.0	BD Biosciences	http://www.bdbiosciences.com/us/instruments/clinical/software/flow-cytometry-acquisition/bd-facsdiva-software/m/333333/overview

### Contact for Reagent and Resource Sharing

Further information and requests for resources and reagents should be directed to and will be fulfilled by the Lead Contact, Alexandra Snyder (snyderca@mskcc.org).

### Experimental Model and Subject Details

#### Human subjects research

Patient samples were collected and analyzed after informed consent to the institutional tissue collection protocol, and approval by the Internal Review Board (IRB) of Memorial Sloan Kettering Cancer Center. The biological sex of the patient is female (XX). The age of the patient at the time the primary sample was resected was 53 years old, and 60 years old at the time the metastatic samples were resected.

#### Distribution and availability of blood and tissue used in this study

The tissue and peripheral blood used for this work are nearly exhausted. Investigators interested in their use should contact the Lead Contact and a Material Transfer Agreement (MTA) put in place as per MSKCC Standard Operating Procedures.

### Method Details

#### Whole exome sequencing

Whole exome sequencing was performed using the Illumina protocol at the Broad Institute of MIT and Harvard, Cambridge, MA, USA. Illumina sequencing of exomes was employed targeting approximately 37.7Mb of mainly exonic territory made up of all targets from Broad Institute’s Agilent exome design (Agilent SureSelect All Exon V2), all coding regions of Gencode V11 genes, and all coding regions of RefSeq gene and KnownGene tracks from the UCSC genome browser (http://genome.ucsc.edu). Data was analyzed using the Broad Picard Pipeline which includes de-multiplexing and data aggregation.

The Illumina exome sequencing uses Illumina’s in-solution DNA probe based hybrid selection method that uses similar principles as the Broad Institute-AgilentTechnologies developed in-solution RNA probe based hybrid selection method (Fisher et al., 2011, Gnirke et al., 2009) to generate Illumina exome sequencing libraries. Pooled libraries were normalized to 2nM and denatured using 0.2N NaOH prior to sequencing. Flow cell cluster amplification and sequencing were performed according to the manufacturer’s protocols using either the HiSeq 2000 v3 or HiSeq 2500. Each run was a 76 bp paired-end with a dual eight-base index barcode read. The sequencing depths of the samples were: normal blood sample (90% at 20X), primary (82% at 50X), spleen (78% at 50X), RUQ (60% at 50X), liver (89% at 50X), and vaginal cuff (77% at 50X) tumors.

#### Gene expression

RNA was extracted from FFPE samples using the RecoverAll Total Nucleic Acid Isolation from Thermo Fisher Scientific (Catalog Number: AM1975). RNA expression was assessed using the human Affymetrix Clariom D Pico assay. Arrays were analyzed using the SST-RMA algorithm in the Affymetrix Expression Console Software. Expression was determined by using the Affymetrix Transcriptome Analysis Console, and for genes displaying inconsistent expression between probes, the *SRY* gene signal was used as a cutoff. LOESS normalization across samples was implemented before differential expression analysis and ssGSEA (Tables S2A and S2C) using:

# R 3.4.0

library(affy) # version 1.54

data_nom<-normalize.loess(data, family.loess=”gaussian”)

#### Immunofluorescent staining

The immunofluorescent staining and cell counting were performed at Molecular Cytology Core Facility of Memorial Sloan Kettering Cancer Center using Discovery XT processor (Ventana Medical Systems) by a cytologist blinded to the sample identifiers and conditions. The tissue sections were deparaffinized with EZPrep buffer (Ventana Medical Systems), antigen retrieval was performed with CC1 buffer (Ventana Medical Systems). Sections were blocked for 30 min with Background Buster solution (Innovex) followed by avidin/biotin blocking for 8 min. Pseudocolors were applied as follows: CD4 A594, FOXP3 A488, CD8 A647; CD68 and CD3 A594 and PD-L1 A488. Cells were detected using the DAPI image, which was processed and segmented using ImageJ/FIJI (NIH). Appropriate threshold values were set for all other markers, and the number of cells with positive signal above the threshold was counted for all single and double staining.

For multiplex staining, each marker was added consecutively in separate staining runs as follows. CD4/FoxP3/CD8: Sections were incubated with anti-CD4 (Ventana, cat#790-4423, 0.5 μg/ml) for 5 hr, followed by 60 min incubation with biotinylated goat anti-rabbit IgG (Vector Laboratories, cat # PK6101) at 1:200 dilution. The detection was performed with Streptavidin-HRP D (part of DABMap kit, Ventana Medical Systems), followed by incubation with Tyramide Alexa 488 (Invitrogen, cat# T20922) prepared according to manufacturer instruction with predetermined dilutions. Next, slides were incubated with anti-FoxP3 (Abcam, cat#ab20034, 5 μg/ml) for 4 hr, followed by 60 min incubation with biotinylated horse anti-mouse IgG (Vector Laboratories, cat# MKB-22258) at 1:200 dilution. The detection was performed with Streptavidin-HRP D (part of DABMap kit, Ventana Medical Systems), followed by incubation with Tyramide Alexa Fluor 568 (Invitrogen, cat# T20914) prepared according to manufacturer instruction with predetermined dilutions. Finally, sections were incubated with anti-CD8 (Ventana, cat#790-4460, 0.07 μg/ml) for 5 hr, followed by 60 min incubation with biotinylated goat anti-rabbit IgG (Vector, cat # PK6101) at 1:200 dilution.

PDL1/CD68 or CD3: First, sections were incubated with anti-PDL1 (Cell Signaling, cat#13684, 5 μg/ml) for 5 hr, followed by 60 min incubation with biotinylated goat anti-rabbit IgG (Vector, cat # PK6101) at 1:200 dilution. The detection was performed with Streptavidin-HRP D (part of DABMap kit, Ventana Medical Systems), followed by incubation with Tyramide Alexa 488 (Invitrogen, cat# T20922) prepared according to manufacturer instruction with predetermined dilutions. Next, slides were incubated with anti-CD68 (DAKO, cat#M0814, 0.02 μg/ml) for 5 hr, followed by 60 min incubation with biotinylated horse anti-mouse IgG (Vector Labs, cat# MKB-22258) at 1:200 dilution, or with anti-CD3 (DAKO, cat#A0452, 1.2 μg/ml) for 4 hr, followed by 60 min incubation with biotinylated horse anti-rabbit IgG (Vector Labs, cat# PK6101) at 1:200 dilution. The detection was performed with Streptavidin-HRP D (part of DABMap kit, Ventana Medical Systems), followed by incubation with Tyramide Alexa Fluor 568 (Invitrogen, cat# T20914) prepared according to manufacturer instruction with predetermined dilutions. After staining slides were counterstained with DAPI (Sigma Aldrich, cat# D9542, 5 μg/ml) for 10 min and coverslipped with Mowiol.

#### Sequenced-based HLA typing

HLA class I and class II 6-digit typing was performed at the New York Blood Center by sequence-based typing and specific sequence primers.

#### TCR sequencing

High-throughput sequencing of the T cell receptors present in the samples and blood of the patient was done using the immunoSEQ assay platform (Adaptive biotechnologies).

#### PBMC–neoepitope assay

The predicted peptides were synthesized (Genscript Corporation). PBMCs were cultured in complete RPMI (Core Media Preparation Facility MSKCC) with peptides at 1 μg/mL, peptide vehicle (DMSO, Sigma-Aldrich) and CEF peptide pool (2 μg/ml, C.T.L) for 21 days with peptide restimulation at day 7 and day 14. IL-2 (Proleukin, Chiron) and IL-15 (Peprotech, cat#200-15) were added every 3 days at 10 IU/mL and 10 ng/mL respectively. Intracellular Cell Staining (ICS) was performed at day 14, and day 21 after 6 hr re-stimulation in the presence of monensin for 5 hr (GolgiStop, BD). Cells were then stained for 15 min with viability dye (LIVE/DEAD Fixable Aqua Dead Cell Stain Kit, ThermoFisher) at 4°C followed by 30 min incubation with CD45-APC-H7 (BD PharMingen, clone 2D1), CD3-Pacific Blue (BD PharMingen, clone UCHT1), CD4-PerCP-Cy5.5 (eBioscience, clone OKT4), CD8-PE (BD Biosciences, clone SK1). Cells were then fixed and permeabilized with BD Cytofix/Cytoperm (BD Biosciences) for 20 min at 4°C and washed with BD Perm/Wash (BD Biosciences). The ICS was performed in BD Perm/Wash with IFN-γ-FITC (eBioscience, clone GZ-4) and TNF-α-PE-Cy (eBioscience, clone MAb11) at 4°C for 30 min. Samples were acquired on a BD LSRII flow cytometer (BD Biosciences) and the analysis was performed on FlowJo software (FlowJo, LLC).

### Quantification and Statistical Analysis

#### Tumor volume calculation

The two axes CT scan measurements and the equation for the ellipsoid volume were used to estimate tumor volumes:

$$V=\frac{4}{3}\pi \times a\times b\times c$$

Where a and b are the two axes and c is their mean.

#### Mutation calling

Reads with mapping quality below 30 in the BAM files were filtered out before mutation calling. Somatic single nucleotide variants (SNVs) were called using MuTect version 1.1.4 (Cibulskis et al., 2013). Identified missense mutations were manually reviewed using the Integrative Genomics Viewer version 2.3.61 (Robinson et al., 2011, Thorvaldsdóttir et al., 2013).

#### Phylogenetic tree inference

The phylogenetic tree was generated as described in Murugaesu et al. (2015). A binary presence/absence matrix of all non-silent mutations was used as input for the R package phangorn version 2.0.2 (Schliep, 2011). UPGMA hierarchical clustering followed by the parsimony ratchet analysis (Nixon, 1999) were implemented to build the unrooted tree, and the acctran function was used to determine branch lengths.

#### Relative copy-number alterations

To extract copy number information based on the sequenced exomes of the samples, CopywriteR version 2.2.0 (Kuilman et al., 2015) was employed in R version 3.2.3. To perform the analysis, mappability information based on the hg19 human reference genome, 20 kb bin size, and default parameters were used.

#### Absolute copy-number alterations and tumor purity

The absolute copy number profiles and the tumor content of the samples were inferred using the computational method ABSOLUTE version 1.0.6 (Carter et al., 2012) in R version 3.2.3. ABSOLUTE integrates segmented copy number data, pre-computed statistical models of recurrent cancer karyotypes, allelic fractions of somatic SNVs, and a probabilistic model framework to jointly estimate candidate tumor purity, ploidy values, absolute copy number data, and subclonal single nucleotide variants (Carter et al., 2012). Tumor purity and absolute copy numbers were obtained using ABSOLUTE default parameters, segmented copy number data derived from CopywriteR, and variant allele frequencies estimated by MuTect (Cibulskis et al., 2013). Best model selection was based on the guidelines provided by GenePattern and the Broad Cancer Genome Analysis group (http://www.broadinstitute.org/cancer/software/genepattern/analyzing-absolute-data). Amplifications and deep deletions were defined as copy-number alterations with at least ± 2 median absolute deviations for each sample copy-number distribution as shown in Figure S1C.

#### Mutation cellular prevalence

Variant allelic cellular prevalence was estimated using PyClone version 0.13.0 (Roth et al., 2014) in Python version 2.7.11. The PyClone pipeline analysis was performed jointly on all samples with their tumor purity and absolute copy number alterations estimated by ABSOLUTE. Total copy number prior probability estimate and the PyClone binomial model were used in the analysis. The mutation variant allele frequencies, closest integer copy number alterations, and tumor purity were used as input. Mutations not present or called in the sample were set to 0. Agglomerative hierarchical cluster analysis with Euclidean distance metric and average linkage clustering was performed on the cellular prevalence values and samples. The *SREBF2*^*S120∗*^ nonsense mutation was not included in the PyClone pipeline because its copy number data was closest to 0.

#### Single-sample gene set enrichment analysis

Single-sample GSEA (Barbie et al., 2009), a modification of standard GSEA (Subramanian et al., 2005), was performed on RNA measurements for each sample using the GSVA package version 1.24.1 (Hänzelmann et al., 2013) in R version 3.3.2 with parameters: method = ‘ssgsea’, and tau = 0.25. Normalized enrichment scores were generated for gene sets belonging to KEGG (Kanehisa and Goto, 2000, Kanehisa et al., 2016) and Reactome (Fabregat et al., 2016). The gene sets were obtained from MSigDB database version 5.2 (Liberzon et al., 2011). In order to identify significantly up- and downregulated gene sets, a *p*-value was calculated for each gene set based on comparison of the enrichment score with 10,000 permutations of randomly sampled gene sets of the same size. All genes listed in the expression array were used to derive the permutated gene sets. Finally, the p values were corrected using Benjamini and Hochberg (BH) method. Enrichment scores were normalized across samples (Tables S2D and S2F) using:

# R 3.4.0

m<-glm(data=data,immunogenicity∼clonality+hla_binding,family=binomial)

#### Immune cell gene-expression signatures

Tumor purity and total immune component in the tumor samples were analyzed using the ESTIMATE algorithm method version 1.0.13 (Yoshihara et al., 2013) on the gene expression data using the option: platform = affymetrix in R version 3.4.0. Then, selection of probes with the highest variance for each gene was performed to deconvolute cell type specific immune signatures. The deconvolution was achieved using CIBERSORT Jar version 1.05 (https://cibersort.stanford.edu/) with the standard LM22 signature gene file, and 1000 permutations to calculate deconvolution p values (Newman et al., 2015).

#### Whole-exome sequencing-based HLA inference

The HLA genotyping algorithms OptiType version 1.0 (Szolek et al., 2014) and POLYSOLVER version 1.0 (Shukla et al., 2015) with default parameters were employed for HLA class I 4-digit inference. POLYSOLVER HLA-I typing and mutation calling were performed using samtools version 0.1.19 and novocraft 3.02.05 for the alignment, and MuTect version 1.1.7 for the variant calling.

#### Neoepitope predictions

##### In silico mutant peptide generation

To predict neoepitopes, “wild-type” petide 17mers (for HLA-I) and 29mers (for HLA-II) with the affected amino acid in the middle for each missense mutation were retrieved from the GRCh37.74 human reference proteome (http://ftp.ensembl.org/pub/release-74/fasta/homo_sapiens/pep/). To generate “mutant” peptides, the affected amino acid was replaced in silico with the corresponding mutant amino acid.

##### HLA class I epitope binding predictions

Mutant peptides were used as input for the T Cell Epitope Prediction Tools included in the Immune Epitope Database and Analysis Resource (IEDB) 3.0 (http://www.iedb.org/) (Vita et al., 2015). The HLA class I epitope binding predictions were performed using the HLA-I IEDB algorithms Consensus (Kim et al., 2012) and the artificial neural network (NetMHC) version 3.4 (Lundegaard et al., 2008, Nielsen et al., 2003) independently yielding same conclusions. For Consensus method – which combines NetMHC, the stabilized matrix method (Peters and Sette, 2005), and the combinatorial peptide libraries method (Sidney et al., 2008) – 9 mers with a relative percentile rank ≤ 1% for each HLA-I allele were considered binders to cover most of the potential immune responses as previously suggested (Kotturi et al., 2007, Moutaftsi et al., 2006). For NetMHC, different cut-off values were evaluated independently and compared between each other. 9mers with absolute IC_50_ affinity values ≤ HLA-I specific cutoffs were considered binders (http://help.iedb.org/entries/23854373) (Paul et al., 2013). HLA-I specific cut-offs were not available for HLA-I C alleles, therefore an IC_50_ ≤ 500nM was used instead. All mutant predicted binders were considered for the analyses, i.e., for each missense mutation, up to six binders for HLA-I (A, B, C alleles) and up to four binders for HLA-II (DQ and DR alleles). Since NetMHC gives actual nM binding affinities, and HLA-I specific cutoffs have been estimated, we used NetMHC predictions throughout the manuscript.

##### HLA class II epitope binding predictions

HLA class II epitope binding predictions on 15mers were performed using the HLA-II IEDB algorithms Consensus (Wang et al., 2008, Wang et al., 2010), NetMHCII version 2.2 (Nielsen and Lund, 2009), and Sturniolo (Sturniolo et al., 1999) since these were the only available methods for the patient HLA-II alleles. The Consensus method used the relative percentile ranks of NetMHCII and Sturniolo, and 15mers with percentile ranks ≤ 1% were considered binders. 15 mers with NetMHCII IC_50_ ≤ 500nM or Sturniolo percentile rank ≤ 1% were considered binders, which are more stringent cut-off values than the IEDB recommended 1000 nM for NetMHCII and ≤ 10% percentile rank for Sturniolo. In the authors’ knowledge, HLA-II specific NetMHCII cut-offs have not been reported.

#### Neoepitope depletion analysis

##### TCGA ovarian cancer null model

To analyze neoepitope depletion across the different samples, we followed the method developed by Rooney and colleagues using only expressed mutations. Commonly mutated genes were not included as indicated (Rooney et al., 2015). The method compares the samples to a data driven null model. To generate the null model and estimate neoepitope depletion, the nucleotide sequences flanking each mutation (context of the mutation) are taken into account, thus 192 possible codon mutations are considered (64codons×3possiblemutations=192possiblechanges). To control for tumor type differences, we used TCGA ovarian cancer samples to generate the null model (Cancer Genome Atlas Research Network, 2011). Context of the mutations for the TCGA ovarian cancer samples and the case study tumor samples were obtained from the assembly of the Genome Reference Consortium Human Reference 37. Only TCGA ovarian cancer samples with mutation context in all missense and silent mutations were included (n = 150). We predicted HLA-I neoepitopes of TCGA ovarian cancer samples using the same approach as for the case study samples described above.

Neoepitope depletion for each sample was calculated as follows. First, the expected number of missense mutations per silent mutation $\left(N_{s}\right)$ and the expected number of predicted neoepitopes per missense mutation $\left(B_{s}\right)$ were calculated using all samples (TCGA ovarian cancer samples and the patient’s samples), where Ns and Bs are vectors with 192 components each:

$$N_{s}=\left(\frac{missense_{AAA\to ACA}}{silent_{AAA\to ACA}},\frac{missense_{AAA\to AGA}}{silent_{AAA\to AGA}},\cdots ,\frac{missense_{TTT\to TGT}}{silent_{TTT\to TGT}}\right)$$

$$B_{s}=\left(\frac{neoepitope_{AAA\to ACA}}{missense_{AAA\to ACA}},\frac{neoepitope_{AAA\to AGA}}{missense_{AAA\to AGA}},\cdots ,\frac{neoepitope_{TTT\to TGT}}{missense_{TTT\to TGT}}\right)$$

Therefore, each component of the vector $N_{s}$ corresponds to the fraction of missense mutations per silent mutation, and each component of the vector $B_{s}$ corresponds to the number of predicted neoepitopes per missense mutation. In both vectors, $N_{s}$ and $B_{s}$, each component corresponds to the ratio of a particular codon change. The components of $N_{s}$ can be computed because the counts of the mutations take into account the three possible reading frames.

Second, the count of silent mutations for each codon change $\left(S_{s}\right)$ was calculated for each sample. Thus, $S_{s}$ is a vector with 192 components where each component is the number of silent mutations with a particular type of codon change for a given sample:

$$S_{s}=\left(silent_{AAA\to ACA},silent_{AAA\to AGA},\cdots ,silent_{TTT\to TGT}\right)$$

Third, the expected number of missense mutations $\left(N_{pred}\right)$ and the expected number of neoepitopes $\left(B_{pred}\right)$ were calculated for each sample.

$$N_{pred}=\sum_{m}^{Silent}S_{s}\left(m\right)\times N_{s}\left(m\right)$$

$$B_{pred}=\sum_{m}^{Silent}S_{s}\left(m\right)\times N_{s}\left(m\right)$$

m∈(AAA→ACA,AAA→AGA,⋯,TTT→TGT)

Where Silent represents the number of silent mutations.

Fourth, the expected and observed numbers of neoepitopes per missense mutation for each sample were calculated as follows:

$$Expected=\frac{B_{pred}}{N_{pred}}$$

$$Observed=\frac{B_{obs}}{N_{obs}}$$

Where $N_{obs}$ is the observed number of missense mutations and $B_{obs}$ the predicted number of neoepitopes for each sample.

Finally, for each sample these ratios were compared and the ratio or observed versus expected neoepitopes calculated:

$$R=\frac{Observed}{Expected}$$

Each sample has a ratio R, thus a distribution of log_2_ ratios is generated as shown in Figure S4C top panel. Empirical two-sided *p*-value thresholds were calculated because the ratios do not follow a normal distribution (Shapiro-Wilk test, D’Agostino-Pearson’s test, and Kolmogorov-Smirnov test). The calculations can be found in Tables S4E–S4G.

##### Permutation null model

To compare the levels of neoepitope depletion only between the patient’s samples, we generated sample specific null models based on 150 random unique permutations (redundant permutations excluded) of the samples and their mutations (Table S4H). The number of permutations was selected based on the number of samples used in the TCGA ovarian cancer neoepitope depletion analysis (n = 150). Permutated and real samples were analyzed together using the same approach as for the TCGA ovarian cancer neoepitope depletion analysis described above. A permutation-based null model for each sample was used to control for the number of mutations. Empirical two-sided *p*-value thresholds were calculated for each distribution because the ratios do not follow a normal distribution (Shapiro-Wilk test, D’Agostino-Pearson’s test, and Kolmogorov-Smirnov test). The calculations can be found in Figure S4C and Table S4I.

#### Immunogenicity predictions

Immunogenic properties of HLA class I epitopes were estimated in silico using the IEDB resource tool “MHC I Immunogenicity” (http://tools.iedb.org/immunogenicity/), which combines the chemical and physical properties of the amino acids, their position in the epitope, and the HLA-I subtype allele to estimate the immunogenicity of a given neoepitope-HLA complex (Calis et al., 2013). To compare clonal and sub-clonal predicted immunogenic properties, we used two approaches:

#### Absolute score comparison

Two-sided Mann-Whitney rank tests were calculated to compare absolute scores between clonal and sub-clonal predicted binders and non-binders. In Figures S5A–S5C, n refers to the number of peptides in each category. The Mann-Whitney rank test was employed because the absolute score distributions do not follow a normal distribution (Shapiro-Wilk test, D’Agostino-Pearson’s test, and Kolmogorov-Smirnov test), and because the number of peptides in each category is different a Wilcoxon signed-rank test could not be calculated.

#### Binomial immunogenicity comparison

Generalized linear models (GLM) were used to compare the probability of a peptide having immunogenic properties or not according to it clonal status and HLA binding affinity. The binomial GLM approach was considered appropriate for this setting because immunogenicity can be considered a binomial process, immunogenic or non-immunogenic. In this scenario, however, the binomial process corresponds to whether an epitope has biochemical properties associated with immunogenicity (score ≥ 0) that outweigh properties associated with no immunogenicity (score < 0). Importantly, predicted immunogenicity scores < 0 can still elicit an immunogenic response, but overall they have less immunogenic properties than positive scores (Calis et al., 2013). To further explain variation in the intrinsic immunogenic predictions we included HLA-I binding affinity (nM) as an explanatory variable. We then calculated the probability of a peptide having a positive immunogenic score or not based on the samples’ neoepitope data. No interaction between clonality and HLA-I binding affinity was found, thus the interaction was excluded from the model. The final binomial GLM formula used is:

# R 3.4.0

m<-glm(data=data,immunogenicity∼clonality+hla_binding,family = binomial)

#### TCR sequencing analysis

Analysis of the sequences was performed on the immunoSEQ ANALYZER 3.0 (Adaptive biotechnologies). T cell rearrangements that are differentially abundant between samples were detected using the Differential Abundance tool by two-sided binomial tests with Benjamini and Hochberg multiple test correction, q value < 0.01 was considered statistically significant.

### Data and Software Availability

Requests for additional data and custom code should be directed to the corresponding authors.

#### Whole-exome sequencing data

The accession numbers for the whole exome sequences reported in this paper are BioSample: SAMN06199513, SAMN06199514, SAMN06199515, SAMN06199516, SAMN06199517, and SAMN06199518.

#### Microarray data

The microarray data discussed in this study have been deposited in NCBI’s Gene Expression Omnibus (Edgar et al., 2002), and the accession number is GEO: GSE92780.

#### TCR sequencing data

The TCR sequencing data discussed in this study will be provided upon request to the Lead Contact in the Data and Software Availability section.

## Author Contributions

Study design, C.A., J.D.W., E.S., A.J.-.S., J.K., T.M., M.L.M., A.S., and O.Z.; Editing manuscript, M.B.G., A.J.-.S., M.L.M., J.R., D.Z., A.S., and H.V.; Analysis, M.B.G., A.J.-.S., H.A.V., and H.V.; Bioinformatics, A.J.-.S.; Data interpretation, A.J.-.S., M.L.M., and A.S.; Pathologic evaluation, Y.L. and K.J.P.; Microscopy, Y.L.; Patient’s medical oncologist, J.K.; ssGSEA analysis, D.M.; Study design related to IF and ICS, T.M.; Design and execution of ICS experiments, S.P.; PD-L1, CD3, and CD68 staining and interpretation, J.R. and D.Z.; Radiology outlines and tumor measurements, H.A.V.; Radiology, H.V.; RNA isolation and expression, T.W.; Patient’s surgeon, O.Z.

## Supplemental Information

Table S1. Mutation Data, Related to Figures 1, 2, 4, S1, S2, S4, and S5 (A) Non-silent mutations in binary format. (B) Mutation cellular prevalence. (C) Log_2_ gene expression of mutations after SST-RMA and LOESS normalizations. (D) Log_2_ gene expression of mutations after SST-RMA normalization. (E) Gene expression of mutations in binary format (see STAR Methods).

Table S2. Gene-Expression and Single-Sample Gene Set Enrichment Analysis Data, Related to Figures 2 and S2 (A) Log_2_ gene expression after SST-RMA and LOESS normalizations. (B) Gene expression in binary format (see STAR Methods). (C) Log_2_ gene expression after SST-RMA. (D) Enrichment scores after *Z* score normalization. (E) Enrichment score q values. (F) Raw enrichment scores. (G) Enrichment scores and q values of significantly differentially enriched pathways. (H) REACTOME immune-system pathway genes. (I) KEGG systemic lupus erythematosus genes. (J) REACTOME T cell-signaling pathway genes. (K) KEGG Wnt-signaling pathway genes.

Table S3. Tumor-Immune Microenvironment Data, Related to Figure 3 (A) ESTIMATE data. (B) CIBERSORT data. (C) Immunofluorescence whole-slide quantification data.

Table S4. HLAs, Neoepitope Prediction, and Neoepitope Depletion Data, Related to Figures 4 and S4 (A) *HLA* genotypes. (B) HLA-I neoepitope binding-affinity predictions. (C) HLA-II neoepitope binding-affinity predictions. (D) Expressed predicted binders. (E) Samples and predicted HLA-I binding affinity of expressed mutations. (F) TCGA ovarian cancer samples and predicted HLA-I binding affinity of expressed mutations. (G) Neoepitope depletion ratio of TCGA ovarian cancer samples and case-study samples. (H) Randomly permutated samples and predicted HLA-I binding-affinity-expressed mutations (see STAR Methods). (I) Neoepitope depletion ratios of randomly permutated samples and real case-study samples (see STAR Methods).

Table S5. TCR Sequencing and T Cell-Neoepitope Challenge Data, Related to Figure 4, 5, S6, and S7 (A) Samples and blood TCR sequencing. (B) Expressed predicted neoepitope features and percentage of reactive circulating CD8^+^ T cells.
