# Supplementary figures and images for: Heterogeneous Tumor-Immune Microenvironments among Differentially Growing Metastases in an Ovarian Cancer Patient
Source: Cell. 2017 Aug 24;170(5):927–938.e20. doi: 10.1016/j.cell.2017.07.025 (PMC5589211; doi:10.1016/j.cell.2017.07.025)

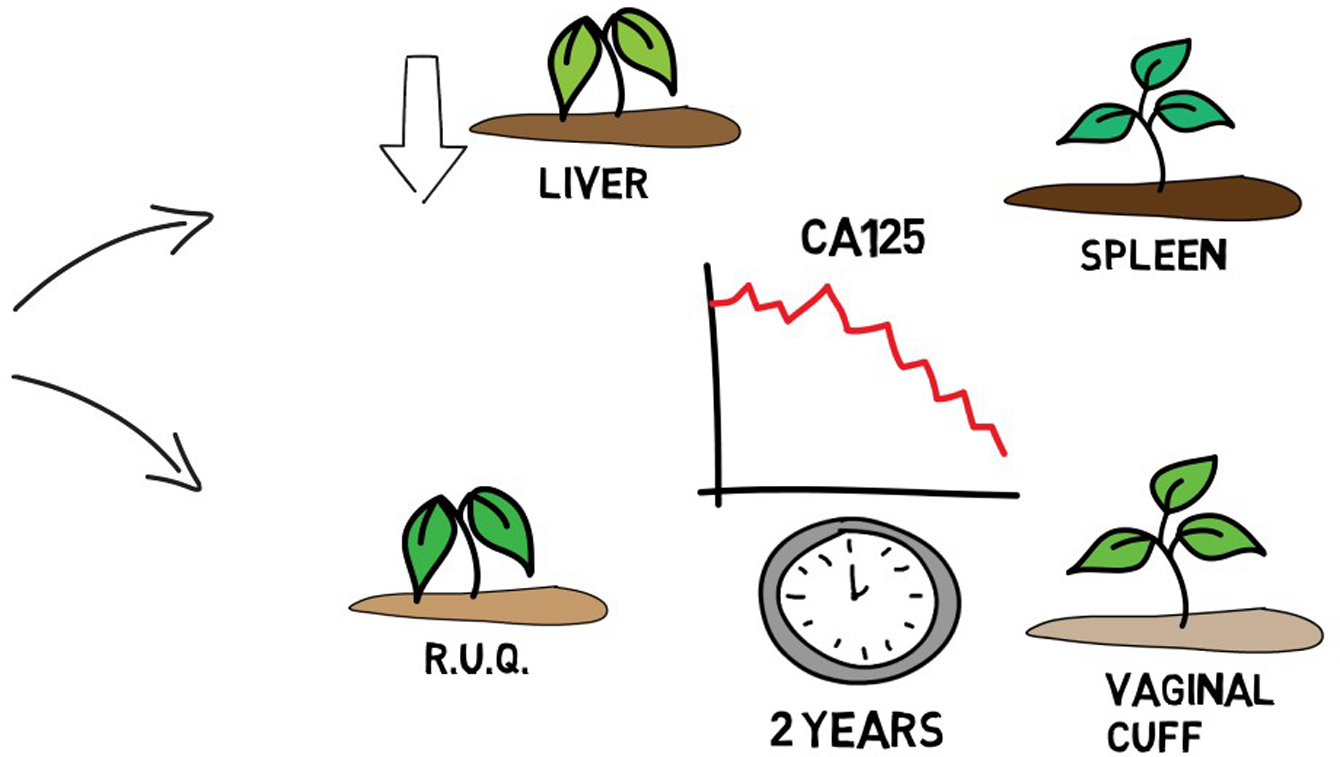

Supplement: Supplementary file 1 [file mmc6.jpg]
